# Phase Equilibria in Systems Involving the Rare-Earth Oxides. Part II. Solid State Reactions in Trivalent Rare-Earth Oxide Systems

**DOI:** 10.6028/jres.064A.031

**Published:** 1960-08-01

**Authors:** S. J. Schneider, R. S. Roth

## Abstract

Selected mixtures in 21 binary and 9 ternary rare-earth oxide systems were studied by X-ray diffraction after heat treatment at 1,650° C and above. Two graphs were drawn to show specific regions of stability for the various structure types. Each gives the average ionic radius of constituent cations versus the mole percent of the smaller cation. One diagram is essentially divided into areas of solid solution of the A, B, and C rare-earth oxide structure types. The other indicates a field of perovskite-type compounds bordered by regions of A, B, or C solid solutions. These diagrams were used to predict the subsolidus phase diagrams of a number of systems. A total of forty-one subsolidus binary and one ternary rare-earth oxide systems were given. A tolerance factor equal to 0.77 was assigned as the minimum value for the formation of a perovskite-type compound.

## 1. Introduction

To date, no comprehensive study has been performed on the phase equilibrium relations between the oxides of the trivalent rare-earth ions. Most of the information available on these materials has centered about studies on the single oxides or on limited portions of systems involving only one trivalent rare-earth oxide. The authors, in a previous study [1],^1^ have reinvestigated the polymorphic characteristics of the individual trivalent rare-earth oxides and essentially confirmed the earlier works of Goldschmidt [2] and of Shafer and Roy [3]. It has been definitely established that at elevated temperatures the stable forms of the rare-earth oxides have the following symmetries: La_2_O_3_, Ce_2_O_3_, Pr_2_O_3_, Nd_2_O_3_—hexagonal; Sm_2_O_3_, Eu_2_O_3_, Gd_2_O_3_—monoclinic; and Tb_2_O_3_, Dy_2_O_3_, Ho_2_O_3_, Er_2_O_3_, Tm_2_O_3_, Yb_2_O_3_, Lu_2_O_3_—cubic. The hexagonal, monoclinic, and cubic forms are referred to as the A, B, and C rare-earth oxide structures, respectively.

Ionic size is probably by far the most important factor in determining the behavior of any mixture of the rare-earth oxides at elevated temperatures. According to Ahrens [4] the radii of the trivalent rare-earth ions are: La^+3^—1.14 A; Ce^+3^—1.07 A; Pr^+3^—1.06 A; Nd^+3^—1.04 A; Sm^+3^—1.00 A; Eu^+3^—0.98 A; Gd^+3^—0.97 A; Tb^+3^—0.93 A; Dy^+3^—0.92 A; Ho^+3^—0.91 A; Er^+3^—0.89 A; Tm^+3^—0.87 A; Yb^+3^—0.86 A; and Lu^+3^—0.85 A.^2^ Because of the relatively small differences between the radii of the various cations, it can be assumed that solid solution would be prevalent in a number of rare-earth oxide systems. In most cases, systems of oxides having like structure types probably form a complete series of solid solutions between the end members. As reported in a previous note [5], compounds occur only in those systems where there is a large difference in size between the constituent cations. These compounds (LaErO_3_, LaTmO_3_, LaYbO_3_, and LaLuO_3_) are all isostructural, having a perovskite-type structure, probably with orthorhombic symmetry.

The purpose of this investigation was to survey the solid state reactions that occur under equilibrium conditions for different mixtures of the trivalent rare-earth oxides and to establish the subsolidus phase equilibrium diagrams for various systems.

## 2. Materials

The oxides used in this study were examined by a general qualitative spectrochemical method and were estimated to contain impurity elements in the following ranges of concentration: La_2_O_3_—0.001–0.01% each Al, Ca, and Si;
0.0001–0.001% each Ag, Cu, and Mg; questionable Pr.Nd_2_O_3_—0.01–0.1% Eu; 0.001–0.01% each
Ho, La, Si, and Yb; 0.0001–0.001% each Ca, Cu, Fe, Lu, Mg, Tm, and Y;
<0.0001% each Ag and Mn; questionable Al, Cr, Er, and Ni.Sm_2_O_3_—0.01–0.1% Dy; 0.001–0.01% each
Gd, La, Si, and Tm; 0.0001–0.001% each Eu, Fe, Ho, Mg, Y, and Yb;
<0.0001% each Ca, Cu, and Mn; questionable Ag, Al, and Ni.Gd_2_O_s_—0.03% Tb; 0.05% Eu; 0.001–0.01% each Cu
and Si; <0.0001% Al.Dy_2_O_3_—0.006% Y; 0.13% Ho; 0.01% Er and Tm; and
0.001–0.01% each Cu and Si.HO_2_O_3_—0.07% Er; 0.001–0.01% Si.Er_2_O_3_—0.001–0.01% each Mn and Si.Tm_2_O_3_—0.01–0.1% each Er and Lu;
0.001–0.01% each Dy, Ho, La, Si, Y, and Yb; 0.0001–0.001% each Ca,
Cu, Fe, and Mg; <0.0001% Ag; and questionable Eu, Gd, and Mn.Yb_2_O_3_—0.001–0.01% each Er, Lu, Si, and Tm;
0.0001–0.001% each Cu, Fe, Mg, and Y; <0.0001% each Ca and Mn; and
questionable Ag and Ce.Lu_2_O_3_—0.01–0.1% Er; 0.001–0.01% each
Dy, Si, Y, and Yb; 0.0001–0.001% each Cu, Mg, and Tm; <0.0001% Ca; and
questionable Ag, Eu, and Mn.

It should be noted that the rare-earth elements do not all have equivalent sensitivities in the spectrochemical method of analysis. It is possible that impurity elements such as Pr, Nd, Sm, Eu, Gd, etc., could be present in some of the above listed oxides in amounts of 0.01 percent or higher and still not be detected.

## 3. Test Methods

Specimens were prepared from either 0.75 or 1.0 g batches of various combinations of the rare-earth oxides. Calculated amounts of each oxide component, corrected for loss due to volatile material, were weighed to the nearest milligram. Each batch was mixed, pressed at 10,000 lb/in.^2^ into a ⅜-in.-diameter pellet, and fired at 1,350° C for a minimum of 6 hr. The specimens were then ground, remixed, again pressed into pellets, and finally fired at 1,650° C for 6 hr. In a number of instances, in order to obtain equilibrium, it was necessary to heat the pellets to 1,900° C for several minutes.

The 1,350° and 1,650° C heat treatments were performed in an air atmosphere using an electrically heated tube furnace wound with 80% Pt–20% Rh wire. An induction furnace having, as the susceptor, a small iridium metal crucible (½-in. diameter by 1116 in. high), was used for the 1,900° C heat treatments.

Equilibrium was considered to have been reached when the X-ray pattern of the specimen showed no change with successive heat treatment or when the X-ray powder data were consistent with the results predicted from a previous set of experiments. All specimens were examined at room temperature by X-ray diffraction with a Geiger-counter diffractometer employing nickel-filtered copper radiation. The various phases were identified by comparison of their X-ray patterns with similar indexed patterns of the pure oxides and perovskite-type compounds.

The boundaries of two-phase areas in a given binary system were determined either by the disappearing-phase method or a variation of the parametric method. The former method consists of studying mixtures which differ in composition only by small increments. The boundaries are established between two adjacent compositions that contain one and two phases, respectively. The parametric method locates the boundaries from a knowledge of the unit-cell parameters of a phase, the average ionic radius and the composition that corresponds to that radius. Roth and Schneider [1] have shown that the unit-cell parameters of the A-, B-, and C-type rare-earth oxides lie on a straight line when plotted against Ahrens’ ionic (cation) radii. According to Vegard’s law, the parameters of solid solutions of these structure types should also plot on the same curve. Once a two-phase area is located by trial and error and the unit cell parameters measured, the average ionic radius of each phase can be determined from the radius-parameter plot. Inasmuch as the unit-cell parameters of the phases in a two-phase area do not change with composition, the boundary compositions can be calculated from the average ionic radius of each phase in any mixture in a two-phase area.

## 4. Results and Discussion

### 4.1. Composition-Stability Diagram

**Figure 1:**
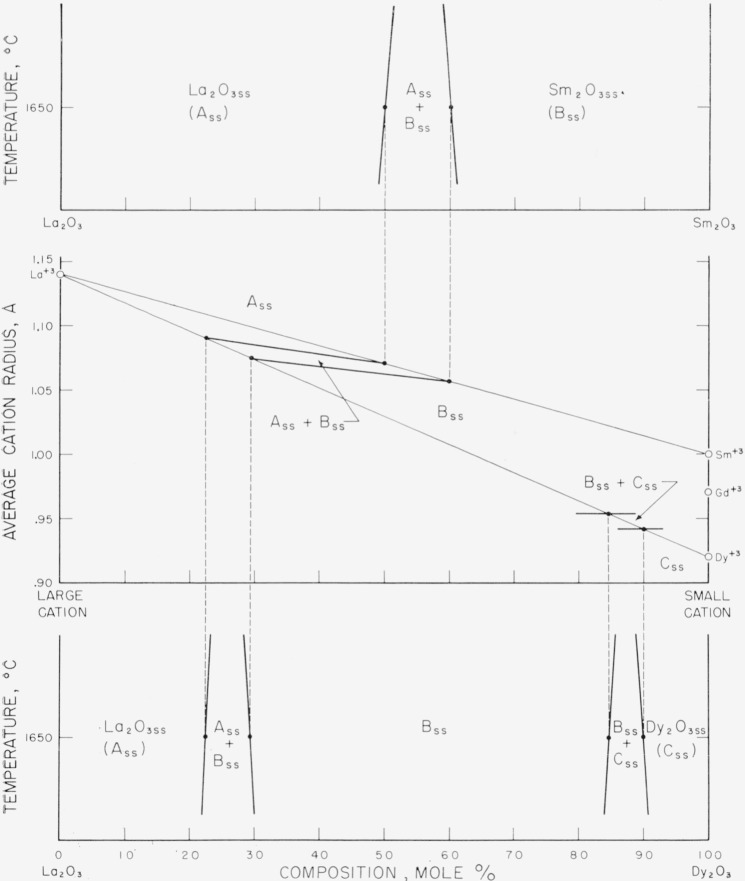
Derivation of a composition-stability diagram from the *La_2_O_3_-Sm_2_O_3_* and *La_2_O_3_-Dy_2_O_3_* systems The exsolution curves of both systems were drawn arbitrarily. The true curvature was not determined.

Because of the great similarity in behavior of different mixtures of the rare-earth oxides, a convenient method was devised whereby the subsolidus phase relations of more than one system could be given on a single diagram. The method selected was a plot of the ionic radii of the constituent cations versus composition. The derivation of a composition-stability diagram is illustrated in figure 1. The center portion of figure 1 shows the plot of the ionic radius of La^+3^ on the left ordinate and the radii of Sm^+3^, Gd^+3^, and Dy^+3^ on the right. The straight lines connecting the radii values represent isothermal cuts through the La_2_O_3_-Sm_2_O_3_ and La_2_O_3_-Dy_2_O_3_ systems. Composition is indicated on the abscissa in the usual manner. For convenience, the radius of the larger cation of any particular combination of oxides was always plotted on the left ordinate. The conventional type subsolidus phase diagrams, as determined in the present study, for the La_2_O_3_-Sm_2_O_3_ and the La_2_O_3_-Dy_2_O_3_ systems are shown in the upper and lower portions of the figure. The two-phase regions of both systems are located on the center diagram by simple projections, in the manner illustrated. The loci of similar boundary points then define the fields of stability for the A_ss_, B_ss_, and C_ss_, structure types for all the intermediate systems (such as the La_2_O_3_-Gd_2_O_3_ system) which are included within the triangle La^+3^-Sm^+3^-Dy^+3^.

Throughout this investigation the equilibrium phases present in a given composition were predicted from rough drafts similar to figure 1 (center) and then investigated experimentally. As more data were accumulated, figure 1 was continually revised and expanded in scope to include all binary systems of La_2_O_3_ with Sm_2_O_3_ and smaller rare-earth oxides. When the predicted and experimental results were in close agreement, the composition-stability diagram for La_2_O_3_ containing binary systems was considered complete.

**Figure 2:**
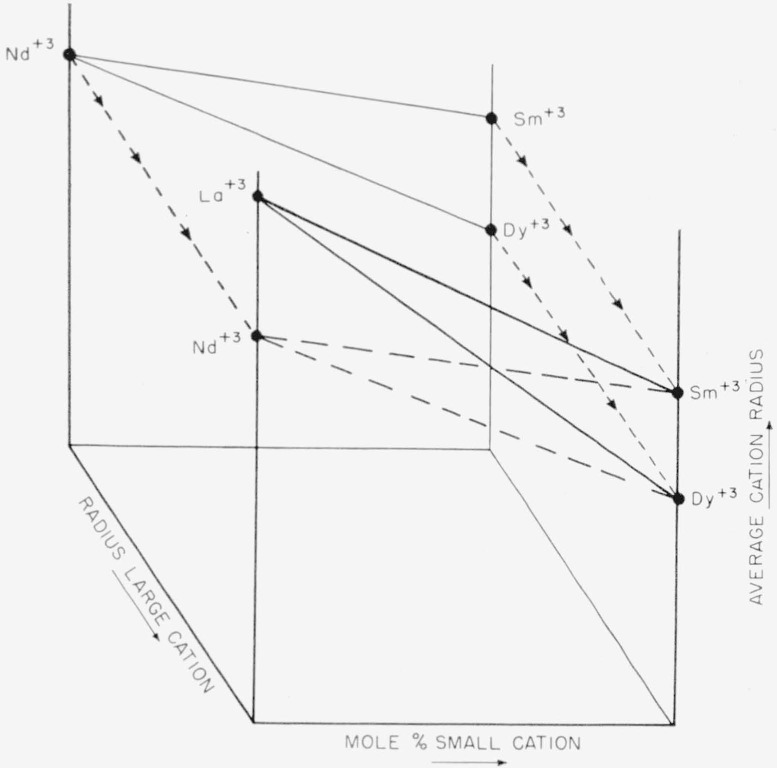
Three-dimensional representation of a composite composition-stability diagram.

In a similar manner, a composition-stability diagram was prepared for sets of binary systems containing Nd_2_O_3_, Sm_2_O_3_, Gd_2_O_3_, etc. Each of these two-dimensional plots represent a plane in a three dimensional figure defined by the orthogonal axes: Radius of large cation, average cation radius of mixture, and composition (mole fraction of small cation). This diagram is illustrated in figure 2. For simplicity only two composition-stability planes are shown. The projection of all of these planes would result in a composite diagram which then defines the phase assemblages for all possible binary systems.

**Figure 3:**
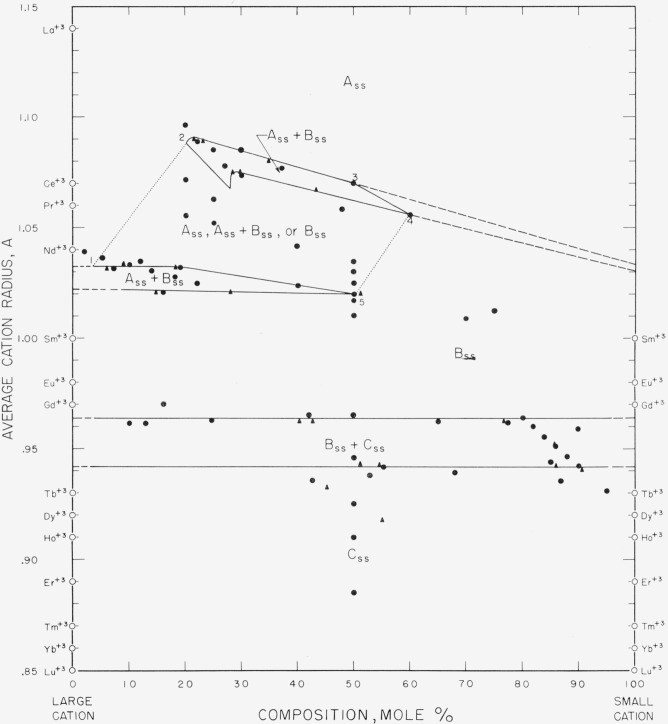
Composition-stability diagram for systems containing only the A, B, or C structure type The diagram is applicable to all systems in which the difference in radii of constituent cations is less than 0.22 A. Area 12345 invalid where radius of large cation >1.04 A and <1.14 A. ●, Compositions studied; ▲, parametric determinations. Guide for predicting the phases present in any multicomponent mixture of oxides of the trivalent rare-earth ions: Locate the average radius of all A-type cations present on the left ordinate and the average radius of all other cations (B and C type) on the right. On the line connecting these points, locate the total mole percentage of cations other than A-type. (Note that A-type cations cannot be plotted on the right ordinate.)If no A-type cations are present, locate the average cation radius of all the B-type cations present on the left ordinate and the average cation radius of all C-type cations on the right. On the line connecting these points, locate the total mole percentage of cations other than B-type.If either A-, B-, or C-type cations are present alone, locate the point on the left ordinate corresponding to the average cation radius.

**Figure 4:**
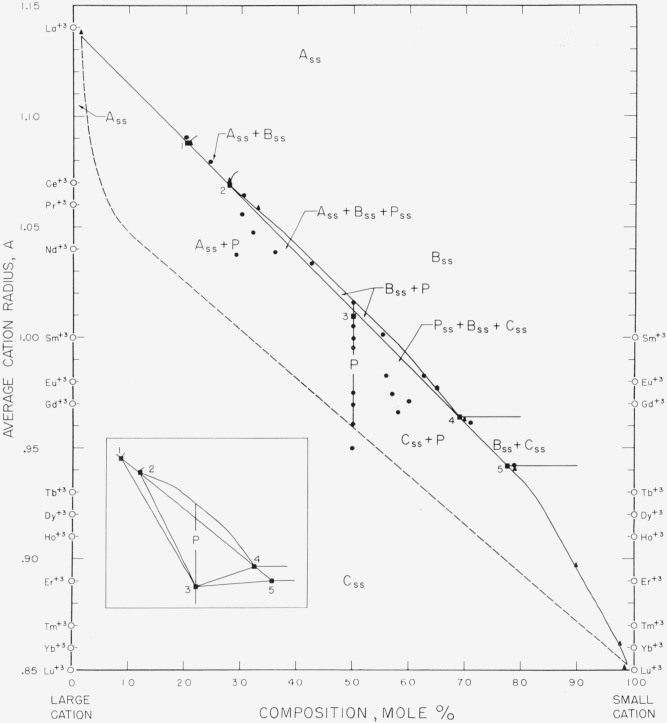
Composition-stability diagram for systems containing perovskite-type compounds The diagram is applicable to all systems in which the difference in radii of constituent cations is equal to or greater than 0.22 A. The inset indicates an exaggerated view of the three-phase areas which are bounded by the triangles 1, 2, 3 and 3, 4, 5. ●, Compositions studied; ▲, parametric determinations. Guide for predicting the phases present in any multicomponent mixture of oxides of the trivalent rare-earth ions: Locate the average radius of all A-type cations present on the left ordinate and the average radius of all other cations (B and C type) on the right. On the line connecting these points, locate the total mole percentage of cations other than A-type. (Note that A-type cations cannot be plotted on the right ordinate.)If no A-type cations are present, locate the average cation radius of all the B-type cations present on the left ordinate and the average cation radius of all C-type cations on the right. On the line connecting these points, locate the total mole percentage of cations other than B-type.If either A-, B-, or C-type cations are present alone, locate the point on the left ordinate corresponding to the average cation radius.

Figures 3 and 4 when superimposed give the completed composite diagram of this general type, as derived from all the experimental data listed in tables 1 and 2. These figures show the fields of stability for the various structure types at room temperature for specimens heat-treated at 1,650° and/or 1,900° C. The boundary lines separating the different fields represent the “best” fit through the several data points as established by the disappearing-phase or the parametric method. Although these two figures ignore such things as possible variations in composition of solid solutions with temperatures, they are useful guides for approximate predictions of the behavior of unknown rare-earth oxide systems at high subsolidus temperatures.

Binary, ternary, and, in theory, four or more component systems are believed to be equally well described by figures 3 and 4. The three guides for predicting the phase relations of any multicomponent system involving oxides of the trivalent rare-earth ions are given in the legends of the figures. It should be emphasized that these rules must be followed implicitly.

Figure 3 pertains only to the A, B, and C structure types. It is applicable to all systems in which the difference between the average radius of the large and small cation is less than 0.22 A. When the difference is greater than this value a perovskite-type compound may form, and figure 4 is then used to predict the phase relationships.

Figure 3 shows two fields of A_ss_+B_ss_ separated by an area in which either A_ss_, A_ss_+B_ss_, or B_ss_ may be present. These fields represent the two phase areas for the La_2_O_3_ and the Nd_2_O_3_ sets of binary systems shown in figure 2. The data showed that this area (1–2–3–4–5 of figure 3) is made up of several A_ss_+B_ss_ fields, the locations of which depend upon the group of systems under consideration. Complete subsolidus phase diagrams cannot be incorporated into figure 3 if A-type cations are involved which have an average radius greater than 1.04 A (Nd^+3^) and less than 1.14 A (La^+3^). This excludes certain portions of systems of A-type oxides or their solid solutions with the B- and C-type rare-earth oxides.

**Figure 5:**
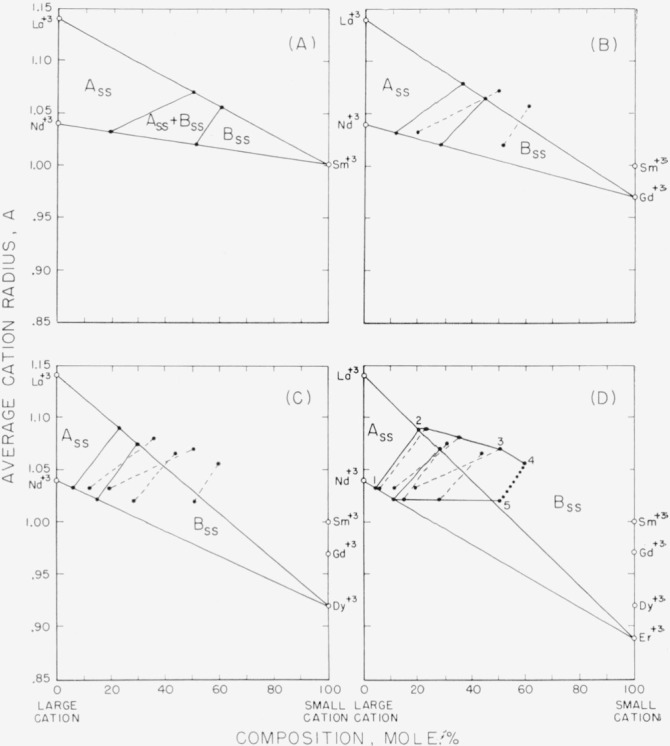
Predicted limits of *A_ss_+B_ss_* area of ternary systems (A) La_2_O_3_-Nd_2_O_3_-Sni_2_O_3_, (B) La_2_O_3_-Nd_2_O_3_-Gd_2_O_3_, (C) La_2_O_3_-Nd_2_O_3_-Dy_2_O_3_, (D) La_2_O_3_-Nd_2_O_3_-Er_2_O_3_. Dashed lines represent boundaries carried over from previous figures. ●, limit of solid solution determined for binary systems. Area 12345 represents same region shown in figure 3

Figure 5 indicates how the A_ss_+B_ss_ field is believed to shift in this region. More experimental data are needed, however, to predict accurately the location and curvature of the boundary lines.

The two triangles formed by joining the points 1, 2, and 3 and points 3, 4, and 5 of figure 4 define the three-phase areas, A_ss_+B_ss_+P_ss_ and P_ss_+B_ss_+C_ss_, respectively. An exaggerated view of these triangles is shown by the inset. These areas are necessary in certain systems of three or more components where three phases in equilibrium are required by the phase rule. Also, the lower dashed portion of the figure should contain three-phase areas, as well as B_ss_+P areas. However, no data were available to confirm this.

### 4.2. B-Type Solid Solutions

The radii of the cations of the B-type oxides vary between fairly narrow limits, 0.97 A for Gd^+3^ to 1.00 A for Sm^+3^. With reference to figure 3, it is evident that in B-type solid solutions, the radii extend to much larger values. The largest average radius of B_ss_ found in the present study is approximately 1.075 A. Mixtures having an average cation radius approaching this value are found in B-type solid solutions in the La_2_O_3_-Dy_2_O_3_ and La_2_O_3_-Ho_2_O_3_ binaries. In multicomponent systems involving La^+3^ and small cations whose average radius is equal to about 0.91 A or 0.92 A, this same radius of 1.075 A can be predicted as the limit of the B_ss_-type field. It is noteworthy that a mixture of an A type (La_2_O_3_) with a C type (Dy_2_O_3_ or Ho_2_O_3_) can give a B-type solid solution that has larger unit-cell dimensions than any mixture of the same A-type oxide with a B-type oxide such as La_2_O_3_ with Sm_2_O_3_.

In general, the B-type solid solutions having the smallest unit-cell dimensions in a given system have approximately equal lattice parameters, regardless of the systems under consideration. These small B-types have average cation radii equal to about 0.964 A and are found in mixtures of either A or B type with C-type oxides. The primary exception found in the present study is in the Sm_2_O_3_-Lu_2_O_3_ system where the B-type solid solution of 1:1 ratio has an average cation radius of about 0.935 A. This solid solution has considerably smaller unit-cell dimensions than the smallest B-type oxide, Gd_2_O_3_, or any other B-type solid solution. Goldschmidt [2] indicated that Dy_2_O_3_ formed a B-type oxide at elevated temperatures, which presumably would have unit-cell dimensions about equal to those of the 1:1 Sm_2_O_3_-Lu_2_O_3_ mixture. The authors [1] however could not make a B-type Dy_2_O_3_^3^.

Both the smallest and largest B-type solid solutions appear to occur in systems where the difference between the radii of the large and small cations (fig. 3) approaches a maximum value. When the difference is at or near a maximum, compound formation is likely. This fact is illustrated by the La_2_O_3_-Er_2_O_3_ [5] and the Sm_2_O_3_-In_2_O_3_ [6] systems where a perovskite-type compound occurs as a stable phase. The difference between the radii of large and small cations in each of these systems is just slightly greater than equivalent values for the La_2_O_3_-Ho_2_O_3_ and the Sm_2_O_3_-Lu_2_O_3_systems.

**Figure 6:**
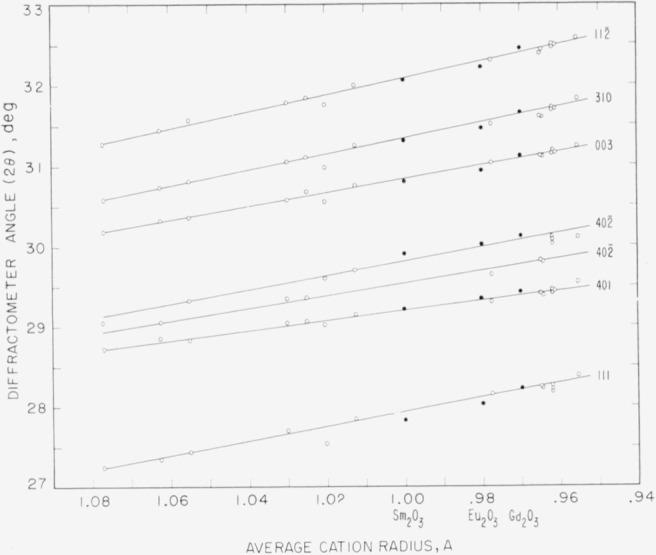
Diffraction angle 2θ of certain X-ray powder lines as a function of average cation radius for *Sm_2_O_3_, Eu_2_O_3_, Gd_2_O_3_*, and B-type solid solutions Indices of each X-ray line are indicated by number adjacent to the corresponding curve. ○, solid solutions;●, pure oxides.

One further correlation can be made between the B-type solid solutions and compound formation. Figure 6 shows a plot of 20 values for several X-ray reflections of different B-type solid solutions versus their corresponding average cation radii. For comparison the appropriate values for Sm_2_O_3_, Eu_2_O_3_, and Gd_2_O_3_ are included. Each reflection is denoted by its respective indices ( $11\bar{2}$, 310, 003, $40\bar{2}$, 401, and 111). The primary anomaly presented by this figure is in the 401 and $40\bar{2}$ separation. The systems fall into two groups having either a narrow or a wide split between the 401 and $40\bar{2}$, dependent upon the mixture under consideration. The narrow split apparently occurs in systems in which a perovskite-type compound might be reasonably expected to occur. These systems for the most part essentially border the perovskite field as shown by figure 4. They are: La_2_O_3_-Dy_2_O_3_, La_2_O_3_-Ho_2_O_3_, La_2_O_3_-Er_2_O_3_, Nd_2_O_3_-Er_2_O_3_, Nd_2_O_3_-Yb_2_O_3_, Nd_2_O_3_-Lu_2_O_3_, and Sm_2_O_3_-Lu_2_O_3_. The one exception to this grouping is the La_2_O_3_-Dy_2_O_3_ system which contains B-type solid solutions of both categories. The narrow split is prevalent throughout most of the La_2_O_3_-Dy_2_O_3_ system. At about the 1:3 La_2_O_3_-Dy_2_O_3_ composition the separation between the 401 and 402 lines widens in a continuous manner until the maximum is reached at the two-phase B_ss_+C_ss_ border. The 401– $40\bar{2}$ split for Sm_2_O_3_, Eu_2_O_3_, and Gd_2_O_3_ is of the wide variety. This is entirely consistent with the other data in that these oxides do not form a perovskite-type compound. Even though all of the B-type solid solutions appear to be isostructural it is suggested that there are in fact two separate varieties, both of which may occur in the same system.

### 4.3. Perovskite-Type Compounds

Figure 4 generally pertains to those systems which contain perovskite-type compounds as stable phases. As mentioned earlier, four compounds of the perovskite type, LaErO_3_, LaTinO_3_, LaYbO_3_, and LaLuO_3_ have been reported [5] in double oxides of the trivalent rare-earth ions. Figure 4 indicates that only one other double oxide, CeLuO_3_, would be expected to form a stable perovskite-type compound. All other stable phases having a perovskite structure would occur in rare-earth oxide systems of three or more components. A stable perovskite-type structure should form in all trivalent rare-earth oxide systems in which the difference between the average radius of the large and small cations is equal to or greater than 0.25 A. This conclusion is substantiated by the La_2_O_3_-Er_2_O_3_, La_2_O_3_-Trn_2_O_3_, La_2_O_3_-Yb_2_O_3_, and La_2_O_3_-Lu_2_O_3_ binary systems. For certain systems such as the La_2_O_3_-Nd_2_O_3_-Lu_2_O_3_ ternary, the minimum difference is decreased to 0.22 A. In essence, when the difference in radii is within the range 0.22 to 0.25 A a stable perovskite-type compound may or may not form. These might be termed border systems and should be studied individually.

Goldschmidt [7] derived a tolerance factor for the perovskite structure which is given by the following formula:

$$t=\frac{R_{a}+R_{o}}{2\left(R_{b}+R_{o}\right)}$$

where

*t*: = tolerance factor
*R_a_*: = ionic radius of larger cation
*R_b_*: = ionic radius of small cation
*R*_O_: = ionic radius of oxygen (= 1.40 A).

As *t* approaches unity the tendency for the formation of a perovskite-type structure becomes greater. The lower limit for the tolerance factor in A^+2^B^+4^O_3_ perovskite structures ranges between 0.71 and 0.77 [6]. No similar minimum values have been assigned to perovskite-type structures in double oxides of the trivalent rare-earth ions. Roth [6] reported *t* values as high as 0.94 for LaA1O_3_ (rhombohedral) and as low as 0.76 for SmInO_3_ (orthorhombic). Apparently 1:1 mixtures of oxides of the trivalent rare-earth ions will always form a stable perovskite-type compound when the tolerance factor is 0.79 or greater. Those mixtures having a *t* value of 0.77 or 0.78 may possibly form such a compound, although it may well be a metastable phase. Based on data in the La_2_O_3_-Nd_2_O_3_-Lu_2_O_3_ system, it is concluded that CeLuO_3_ is a stable perovskite-type compound. The 1:1 mixture of La_2_O_3_ and Ho_2_O_3_ did not form a perovskite compound, even as a metastable phase. Yet each of these mixtures has a *t* value of 0.78. It is possible that a perovskite-type phase may form in ternary mixtures having a tolerance factor lower than the minimum value for binary systems. Although there remain several apparent inconsistencies, a tolerance factor of 0.77 is probably the lowest possible value for a 1:1 rare-earth oxide mixture to form a perovskite-type structure.

Neither radius difference nor tolerance factor gives satisfactory criteria for determining the boundary of the perovskite field in figure 4. The difficulty lies perhaps in the inaccuracy of the ionic radius values. Ahrens’ radii used in this paper are admittedly not entirely correct. However, other values such as those reported by Templeton and Dauben [8] do not fit the data any better. Differences in the thermal expansion characteristics and partially covalent character of the bonds are both important factors to be considered.

### 4.4. Phase Diagrams

**Figure 7:**
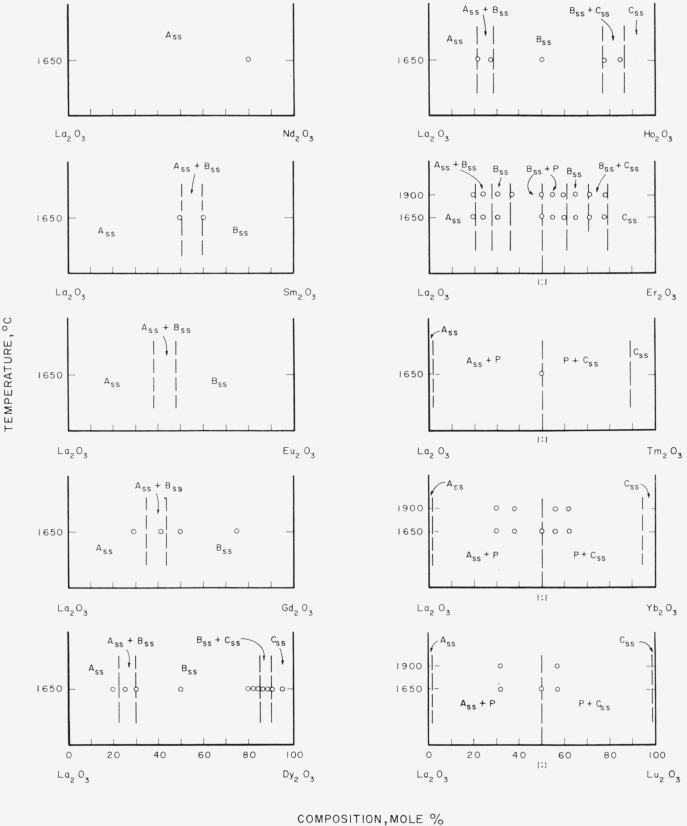
Predicted subsolidus phase diagrams for *La_2_O_3_*-rare-earth oxide binary systems Circles indicate compositions studied. Diagrams applicable only for indicated temperatures.

Figures 7 through 12 give subsolidus phase diagrams for various binary systems involving the trivalent rare-earth oxides. In many instances, the diagrams were completely estimated from either figure 3 or figure 4. When actual experimental data were available (tables 1 and 2), the diagrams, or portions thereof, were drawn to fit these data. Data points are indicated by circles on the diagrams. On the whole, the diagrams must be considered as predicted and for that reason are shown by dashed lines. The general appearance of each diagram is probably correct even though the positions of the boundary lines may be slightly in error. Any possible variations of solid solubility with temperature have been ignored in this work. [Fig f8-jresv64an4p317_a1b] [Fig f9-jresv64an4p317_a1b] [Fig f10-jresv64an4p317_a1b] [Fig f11-jresv64an4p317_a1b]

**Figure 9 f9-jresv64an4p317_a1b:**
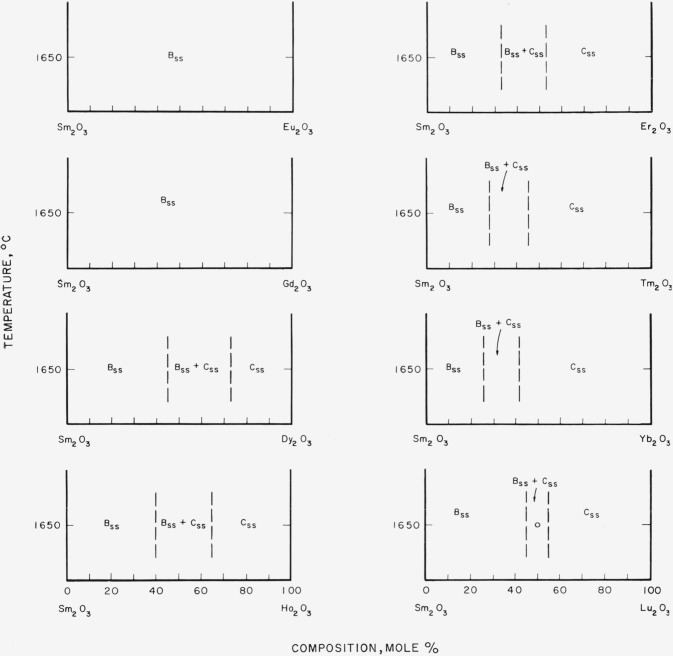
Predicted subsolidus phase diagrams for *Sm_2_O_3_*-rare-earth oxide binary systems Circles indicate compositions studied. Diagrams applicable only for indicated temperatures.

Of all the binary systems given, the Sm_2_O_3_-rare-earth oxide group, figure 9, is most in doubt. The position of the two-phase area, B_ss_+C_ss_, for the Sm_2_O_3_-Lu_2_O_3_ system is unexpectedly shifted to the right (lower radii) from the position predicted by figure 3. If the other systems of this group behave in a consistent manner, the position of this two-phase region should also be located at somewhat lower percentages of Sm_2_O_3_.

**Figure 12:**
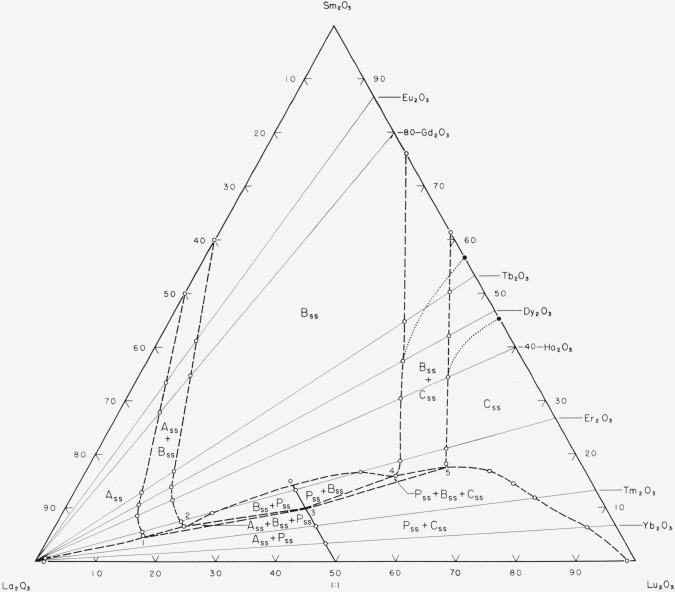
Predicted subsolidus phase diagram for the *La_2_O_3_-Sm_2_O_3_-Lu_2_O_3_* ternary system.

Figure 12 gives a model ternary system, La_2_O_3_-Sm_2_O_3_-Lu_2_O_3_ as predicted by experimental data from rare-earth oxide systems. It was drawn by plotting the boundary points (circles) of binary systems having La_2_O_3_ as one end member. These binary systems are indicated by the joins (solid lines) between La_2_O_3_ and the other listed oxides. The dotted portion shows the position of the two-phase area, B_ss_+C_ss_, that conforms to the data found for the Sm_2_O_3_-Lu_2_O_3_ system. It should be noted that the position of the intersection of the two three-phase areas and the P_ss_ line (point 3) is only estimated. It may lie anywhere along that line. No data were available to fix its position.

It should be emphasized that figure 12 is simply another way of plotting the experimental data listed in tables 1 and 2, which pertain primarily to binary systems. It seems likely that the true La_2_O_3_-Sm_2_O_3_-Lu_2_O_3_ ternary system would have the same general appearance as figure 12, although differing in certain details. In binary systems the B_ss_ field has been observed to occur throughout a wider range of average radii than the equivalent values of the B-type single oxides. Furthermore, the B_ss_ in the Sm_2_O_3_-Lu_2_O_3_ system extends to lower average radii than found in most other binary rare-earth oxide systems. Therefore, it is suggested that the B_ss_ field in figure 12 may extend in the true ternary to even wider ranges than are indicated by the dashed lines. In addition, the limit of the perovskite field may be expected to increase towards higher Sm_2_O_3_ content (lower tolerance factors).

## 5. Summary

A survey was made of the subsolidus reactions that occur in various systems involving oxides of the trivalent rare-earth ions. Mixtures in 21 binary and 9 ternary systems were investigated. Specimens were heat treated at 1,650° C or above and examined at room temperature by X-ray powder diffraction.

On the basis of the survey, two composite composition-stability diagrams were drawn. These are plots of the average ionic radii of the constituent cations versus mole percent of the smaller cation. A join between left and right ordinate values represents an isotherm for the binary system whose cations have radii corresponding to the two limiting ordinates. The diagram may be also applied to some multicomponent systems, with the limiting ordinates representing the average ionic radii of the large and small cations present.

One composition-stability diagram is divided into fields of A_ss_, B_ss_, and C_ss_ oxide structure types with appropriate two-phase areas. This diagram is applicable to all systems in which the difference between the radii of the large and small cations is less than 0.22 A. The other diagram shows a large field of perovskite-type compounds bordered by regions of A_ss_, B_ss_, or C_ss_. The data indicated that a perovskite-type compound always occurred in 1:1 mixtures having a tolerance factor equal to or greater than 0.79. A tolerance factor of 0.77 appears to be the lowest possible value for even a metastable perovskite-type phase to form. In addition, B-type solid solutions apparently can be grouped according to the amount of separation of the 401– $40\bar{2}$ doublet in the X-ray powder pattern.

The composition-stability diagrams were used to predict a number of the subsolidus phase diagrams of the 41 binary and 1 ternary rare-earth oxide systems which are given.

## Figures and Tables

**Figure 8 f8-jresv64an4p317_a1b:**
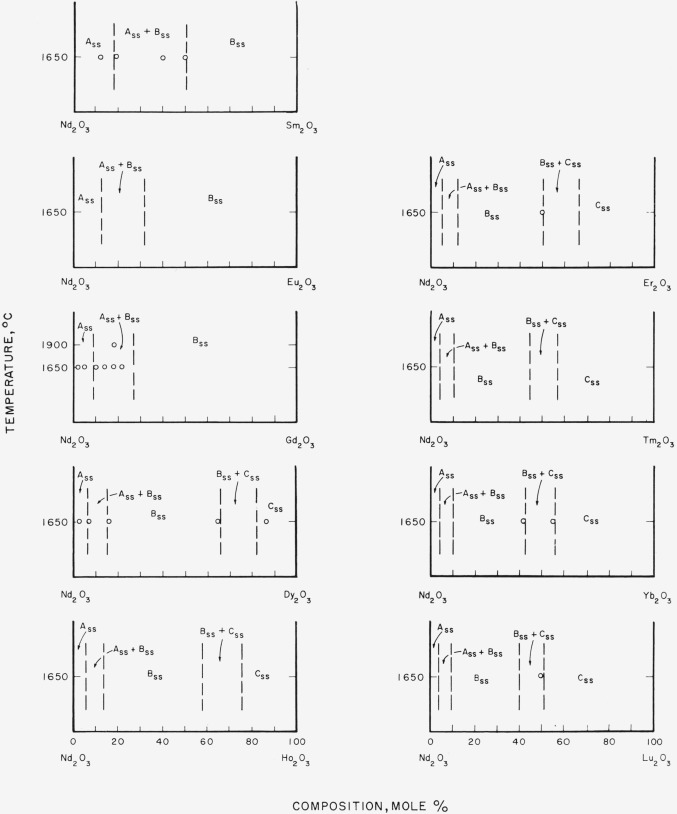
Predicted subsolidus phase diagrams for *Nd_2_O_3_*-rare-earth oxide binary systems Circles indicate compositions studied. Diagrams applicable only for indicated temperatures.

**Figure 10 f10-jresv64an4p317_a1b:**
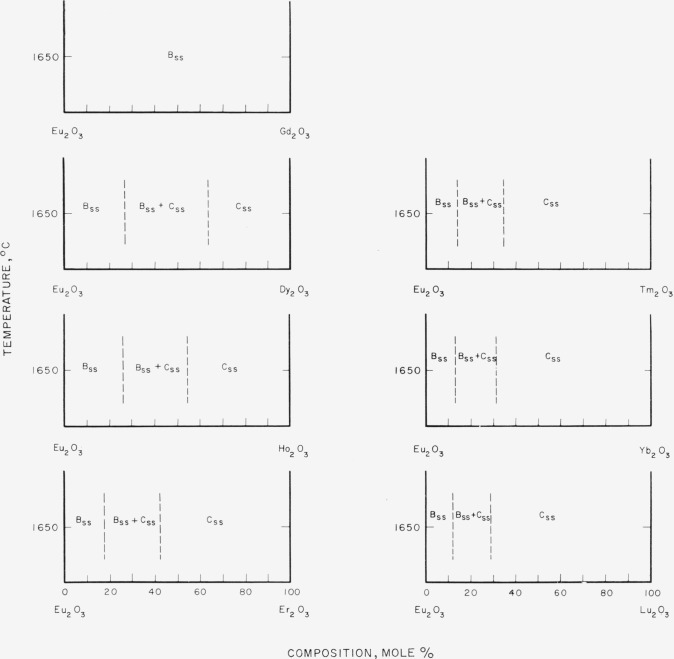
Predicted subsolidus phase diagrams for *Eu_2_O_3_*-rare-earth oxide binary systems Diagrams applicable only for indicated temperatures.

**Figure 11 f11-jresv64an4p317_a1b:**
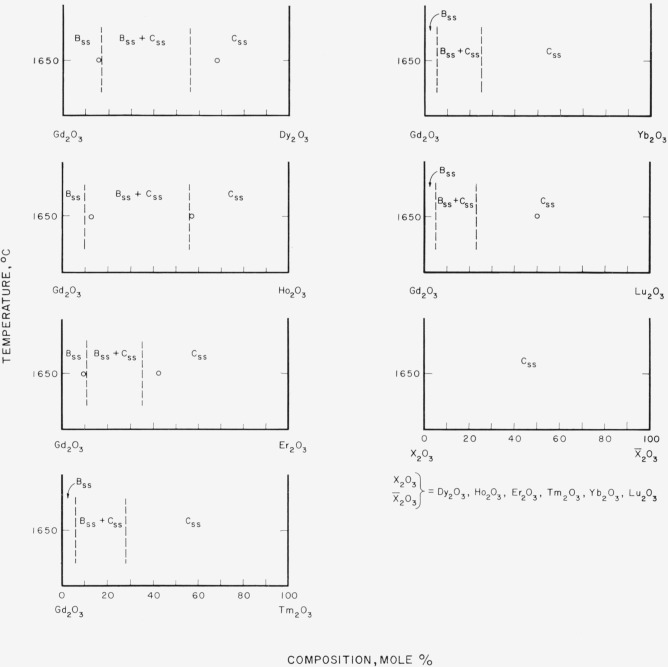
Predicted subsolidus phase diagrams for *Gd_2_O_3_*-rare-earth oxide binary systems and for binary systems involving only C-type rare earth oxides Circles indicate compositions studied. Diagrams applicable only for indicated temperatures.

**Table 1:** Mixed oxide systems formina the A-, B-, or C-structure types

System	Specimen composition	Average radius^a^			Tolerance factor, *t*	Heat treatment		Phases identified by X-ray diffraction^b^	Remarks
		Large cation	Small cation	Mixture		Temp	Time		

	*Mole %*	*A*	*A*	*A*		°*C*	*hr*		
La_2_O_3_-Nd_2_O_3_	20:80	1.14	1.04	1.060	……….	1,650	6	A_ss_	
La_2_O_3_-Sm_2_O_3_	50:50	1.14	1.00	1.070	0.75	1,650	6	A_ss_	
	40:60	1.14	1.00	1.056	……….	1,650	6	B_ss_	
La_2_O_3_-Gd_2_O_3_	70.6:29.4	1.14	.97	1.090	……….	1,650	6	A_ss_	
	63.5:36.5	1.14	.97	1.078	……….	1,650	6	A_ss_+B_ss_	B_ss_ phase present in small amounts.
	50:50	1.14	.97	1.055	.76	1,650	6	B_ss_	
	25:75	1.14	.97	1.013	……….	1,650	6	B_ss_	
La_2_O_3_-Dy_2_O_3_	80:20	1.14	.92	1.096	……….	1,650	6	A_ss_	
	75:25	1.14	.92	1.085	……….	1,650	6	A_ss_+B_ss_	B_ss_ phase present in small amounts.
	70:30	1.14	.92	1.074	……….	1,650	6	B_ss_	
	50:50	1.14	.92	1.030	.77	1,650	6	B_ss_	
	20:80	1.14	.92	. 964	……….	1,650	6	B_ss_	
	18:82	1.14	.92	. 960	……….	1,650	6	B_ss_	
	16:84	1.14	.92	.955	……….	1,650	6	B_ss_	
	14:86	1.14	.92	.951	……….	1, 650	6	B_ss_+C_ss_	
	12:88	1.14	.92	.946	……….	1,650	6	B_ss_+C_ss_	
	10:90	1.14	.92	.942	……….	1,650	6	B_ss_+C_ss_	
	5:95	1.14	.92	.931	……….	1,650	6	C_ss_	
La_2_O_3_-Ho_2_O_3_	78.3:21.7	1.14	.91	1.090	……….	1,650	6	A_ss_+B_ss_	B_ss_ phase present in small amounts.
	73:27	1.14	.91	1.078	……….	1,650	6	A_ss_+B_ss_	
	50:50	1.14	.91	1.025	.78	1, 650	6	B_ss_	
	22.6:77.4	1.14	.91	.962	……….	1,650	6	B_ss_+C_ss_	
	14.8:85.2	1.14	.91	.944	……….	1,650	6	B_ss_+C_ss_	
Nd_2_O_3_-Sm_2_O_3_	88:12	1.04	1.00	1.035	……….	1,650	6	A_ss_	
	81:19	1.04	1.00	1.032	……….	1,650	6	A_ss_+B_ss_	B_ss_ phase present in smal amounts.
	60:40	1.04	1.00	1.024	……….	1,650	6	A_ss_+B_ss_	
	50:50	1.04	1.00	1.020	.72	1,650	6	A_ss_+B_ss_	
Nd_2_O_3_-Gd_2_O_3_	98:2	1.04	.97	1.039	……….	1,650	6	A_ss_	
	95:5	1.04	.97	1.037	……….	1,650	6	A_ss_	
	90:10	1.04	.97	1.033	……….	1,650	6	A_ss_	Pattern of pellet surface indicated A_ss_+B_ss_+C_ss_; B_ss_+C_ss_ phase nonequilibrium.
	86:14	1.04	.97	1.030	……….	1,650	6	A_ss_+B_ss_	
	82:18	1.04	.97	1.027	……….	1,650	6	A_ss_+B_ss_	
						1,900	.083	A_ss_+B_ss_	
	78:22	1.04	.97	1.025	……….	1,650	6	A_ss_+B_ss_	
Nd_2_O_3_-Dy_2_O_3_	97:3	1.04	.92	1.036	……….	1,650	6	A_ss_	
	93:7	1.04	.92	1.032	……….	1,650	6	A_ss_+B_ss_	
	84:16	1.04	.92	1.021	……….	1,650	6	B_ss_	
	35:65	1.04	.92	.962	……….	1,650	6	B_ss_	
	13.3:86.7	1.04	.92	.936	……….	1,650	6	C_ss_	
Nd_2_O_3_-Er_2_O_3_	50:50	1.04	.89	.965	.75	1,650	6	B_ss_	
Nd_2_O_3_-Yb_2_O_3_	58:42	1.04	.86	.964	……….	1,650	6	B_ss_	
	45:55	1.04	.86	.941	……….	1,650	6	B_ss_+C_ss_	
Nd_2_O_3_-Lu_2_O_3_	50:50	1.04	.85	.945	.77	1,650	6	B_ss_+C_ss_	
Sm_2_O_3_-Lu_2_O_3_	50:50	1.00	.85	.925	.75	1,650	6	B_ss_+C_ss_	Average cation radius of B_ss_~ 0.935A.
Gd_2_O_3_-Dy_2_O_3_	84:16	.97	.92	.962	……….	1,650	6	B_ss_	
	32:68	.97	.92	.936	……….	1,650	6	C_ss_	
Gd_2_O_3_-Ho_2_O_3_	86.7:13.3	.97	.91	.962	……….	1,650	6	B_ss_+C_ss_	C_ss_ phase present in small amounts.
	43.3:56.7	.97	.91	.936	……….	1,650	6	C_ss_	
Gd_2_O_3_-Er_2_O_3_	90:10	.97	.89	.962	……….	1,650	6	B_ss_	
	57.5:42.5	.97	.89	.936	……….	1,650	6	C_ss_	
Gd_2_O_3_-Lu_2_O_3_	50:50	.97	.85	.910	.75	1,650	6	C_ss_	
Dy_2_O_3_-Lu_2_O_3_	50:50	.92	.85	.885	……….	1,650	6	C_ss_	
La_2_O_3_-Nd_2_O_3_-Sm_2_O_3_	24:56:20	1.07	1.00	1.056	……….	1,900	.167	A_ss_	No 1,650° C heat.
	22.5:52.5:25	1.07	1.00	1.053	……….	1,900	.333	A_ss_	No 1,650° C heat.
	18:42:40	1.07	1.00	1.042	……….	1,650	6	A_ss_+B_ss_	
	15:35:50	1.07	1.00	1.035	.73	1,650	6	B_ss_	
La_2_O_3_-Nd_2_O_3_-Gd_2_O_3_	18:12:70	1.10	.97	1.009	……….	1,650	6	B_ss_	
La_2_O_3_-Nd_2_O_3_-Dy_2_O_3_	56:24:20	1.11	.92	1.072	……….	1,650	3	A_ss_	
	52.5:22.5:25	1.11	.92	1.063	……….	1,650	3	A_ss_+B_ss_	
La_2_O_3_-Dy_2_O_3_-Er_2_Os	50:8.3:41.7	1.14	.895	1.018	.78	1,900	.167	B_ss_	No 1,650° C heat.
						1,900	.333	B_ss_	
Nd_2_O_3_-Gd_2_O_3_-Sm_2_O_3_	50:33.3:16.7	1.04	.98	1.010	.73	1,650	3	B_ss_	No 1,650° C heat.
Nd_2_O_3_-Gd_2_O_3_-Dy_2_O_3_	10:54:36	1.04	.95	.959	……….	1,650	3	B_ss_	
Sm_2_O_3_-Gd_2_O_3_-Ho_2_O_3_	25:50:25	.98	.91	.963	……….	1,900	.167	B_ss_	No 1,650° C heat.

a Based on radii given by Ahrens [4].

b A = hexagonal A-type rare-earth oxide structure, B=monoclinic B-type rare-earth oxide structure, C=cubic C-type rare-earth oxide structure.

**Table 2:** Mixed oxide systems forming perovskite-type compounds.

System	Specimen composition	Average radius a			Tolerance factor, *t*	Heat treatment		Phases identified by X-ray diffraction b	Remarks
		Large cation	Small cation	Mixture		Temp	Time		

	*Mole%*	*A*	*A*	*A*		°*C*	*min*		
La_2_O_3_-Er_2_O_3_	80:20	1.14	0.89	1.090	……………	1,650	360	A_ss_+B_ss_	Nonequilibrium.
						1,900	5	A_ss_	
	75.2:24.8	1.14	.89	1.078	……………	1,650	360	A_ss_+B_ss_+P	Nonequilibrium.
				……………	……………	1,900	5	A_ss_+B_ss_	
	69.31	1.14	.89	1.063	……………	1,650	360	B_ss_+P	Nonequilibrium.
				……………	……………	1,900	5	B_ss_	
	63:37	1.14	.89	1.048	……………	1,900	5	B_ss_+P	No X-ray pattern for 1,650° C heat.
	57:43	1.14	.89	1.033	……………	1,650	360	P	Second phase present, probably B_ss_.
						1,900	5	B_ss_+P	
	50:50	1.14	.89	1.015	0.79	1,650	360	P	
						1,900	6	P	
						1,900	30	P	
	45:55	1.14	.89	1.003	……………	1,650	360	B_ss_+P	
						1,900	5	B_ss_+P	
	40:60	1.14	.89	0.990	……………	1,650	360	B_ss_+C_ss_	Nonequilibrium.
						1,900	5	B_ss_+C_ss_+P	Nonequilibrium.
						1,900	16	B_ss_+P	
	35:65	1.14	.89	.978	……………	1,900	5	B_ss_+C_ss_	Nonequilibrium, no. 1650° C heat
						1,900	22	B_ss_+C_ss_	Amount of C_ss_ phase reduced relative to previous 1,900° C heat—C_ss_ nonequilibrium.
	28.8:71.2	1.14	.89	.962	……………	1,650	360	C_ss_+P	Nonequilibrium.
						1,900	6	B_ss_+C_ss_	
	21.6:78.4	1.14	.89	.944	……………	1,650	360	C_ss_+P	Nonequilibrium.
						1,900	6	B_ss_+C_ss_	
La_2_O_3_-Tm_2_O_3_	50:50	1.14	.87	1.005	.79	1,650	360	P	
La_2_O_3_-Tm_2_O_3_	70:30	1.14	.86	1.056	……………	1,650	180	A_ss_+P	
						1,900	5	A_ss_+P	
	62:38	1.14	.86	1.034	……………	1,650	180	A_ss_+P	
						1,900	5	A_ss_+*P*	
	50:50	1.14	.86	1.000	.80	1,650	360	P	
	44:56	1.14	.86	.983	……………	1,650	180	C_ss_+P	
						1,900	5	C_ss_+P	
	38:62	1.14	.86	.966	……………	1,650	180	C_ss_+P	
						1,900	5	C_ss_+P	
La_2_O_3_-Lu_2_O3	68:32	1.14	.85	1.047	……………	1,650	180	A_ss_+P	
						1,900	5	A_ss_+P	
	50:50	1.14	.85	0.995	.08	1,650	360	P	
	43:57	1.14	.85	.975	……………	1,650	180	C_ss_+P	
						1,900	5	C_ss_+P	
La_2_O_3_-Nd_2_O_3_-Yb_2_O3	49:21:30	1.11	.86	1.035	……………	1,900	10	A_ss_+P	No 1,650° C heat.
La_2_O_3_-Nd_2_O_3_-Lu_2_O3	30:20:50	1.10	.85	0.975	.79	1,900	5	P	No 1,650° C heat.
	25:25:50	1.09	.85	.970	.78	1,900	5	P+C_ss_	No 1,650° C heat, nonequilibrimn.
						1,900	15	P+C_ss_	No 1,650° C heat, nonequilibrium, amount of C_ss_ phase reduced relative to previous heat.
	15:35:50	1.07	.85	.960	.78	1,650	360	P	
	10:40:50	1.06	.85	.955	.77	1,650	360	P+B_ss_+C_ss_	Nonequilibrium.
						1,900	10	P+B_ss_+C_ss_	Nonequilibrium, amount of P phase reduced relative to previous heat.
						1,960	10	B_ss_+C_ss_	
	5:45:50	1.05	.85	.950	.77	1,650	360	P+B_ss_+C_ss_	P phase probably nonequilibrium.

a Based on radii given by Ahrens [4].

b A=hexagonal A-type rare earth oxide structure, B=monoclinic B-type rare earth oxide structure, C=cubic C-type rare earth oxide structure, P=orthorhombic distorted perovskite type compound.

## Footnotes

1 Figures in brackets indicate the literature references at the end of this paper.

2 These values are used throughout this paper.

3 A 1:1 mixture of Dy_2_O_3_ and In_2_O_3_ after heat treatment at 1,650° C showed a mixture of B_ss_+C_ss_, with the B_ss_ having parameters equivalent to an average cation radius of about 0.88 A. This is the smallest average radius for B-type solid solution yet reported. It extends well into the C_ss_ region of figure 3.
